# A Novel Variant in the AGPS Gene Causes the Rare Rhizomelic Chondrodysplasia Punctata Type 3: A Case Report

**DOI:** 10.7759/cureus.20543

**Published:** 2021-12-20

**Authors:** Aiman M Shawli, Abdulaziz T Nazer, Yasir Khayyat, Mohammed G Alqurashi, Fahad Hakami

**Affiliations:** 1 General Pediatrics and Pediatric Genetics, King Abdulaziz Medical City Jeddah, Jeddah, SAU; 2 Internal Medicine, King Saud Bin Abdulaziz University for Health Sciences College of Medicine, Jeddah, SAU; 3 Pathology and Laboratory Medicine/Genetics, King Abdulaziz Medical City Jeddah, Jeddah, SAU

**Keywords:** rhizomelic chondrodysplasia punctata type 3, alkylglycerone phosphate synthase, autosomal recessive disorders, peroxisomal disorders, rhizomelic chondrodysplasia punctata

## Abstract

Rhizomelic chondrodysplasia punctata (RCDP) is a devastating medical condition for patients and their families. It is a rare peroxisomal autosomal recessive disorder. It was recognized clinically with skeletal abnormalities and intellectual disabilities mainly due to plasmalogen deficiency. Here, we report a case of a 16-day-old girl who was referred to King Abdulaziz Medical City Jeddah, Saudi Arabia because of dysmorphic features. Her growth parameters were below the 3rd centile with short proximal long bones and multiple joint contractures in the extremities. The radiographs showed rhizomelic and shortening of both humeri and femurs. Moreover, punctate ossification was identified in the upper spine, humeri around the shoulders, and femurs around the knees. We observed other classical features, and the genetic testing confirmed the diagnosis of RCDP type 3. Although RCDP is a rare condition, it is a distressing burden necessitating early diagnosis and a holistic approach for management.

## Introduction

There are plenty of inheritable diseases worldwide affecting the lives of many individuals in their infancy, childhood, and even adulthood; one of which is rhizomelic chondrodysplasia punctata (RCDP). RCDP is a rare peroxisomal disorder that is autosomal recessive. This disorder intervenes with the normal development of many parts of the body. The key features of this condition are skeletal abnormalities, distinguishing facial features, intellectual disability, and respiratory problems. It is a rare inherited disorder with a prevalence of only one in 100,000 individuals [1,2]. RCDP is a peroxisomal disorder causing plasmalogen deficiency, which directly affects bone growth causing bone growth failure that is proportional to the degree of plasmalogen deficiency [3]. There are three types of RCDP, namely, type 1 (RCDP1), type 2 (RCDP2), and type 3 (RCDP3), caused by genetic abnormalities in the *PEX7*, *GNPAT*, and *AGPS* genes, respectively. Genetic testing is needed to differentiate between these three RCDP subtypes because they are indistinguishable clinically. Recently, two more subtypes were described, type 4 (RCDP4) and type 5 (RCDP5), attributed to the *FAR1* and *PEX5* genes, respectively [4]. To the best of our knowledge, only nine RCDP3 cases were reported in the literature with nine pathogenic variants identified in the *AGPS* gene. Of these variants, nine were missense, and two were frameshift [5-9]. Due to this limited number of described cases, it is not currently possible to predict the severity of the disease based on the type of the variant. This report discusses a rare case of RCDP3 caused by a novel variant in *AGPS*. To date, this variant is the third reported pathogenic frameshift variant in *AGPS*.

## Case presentation

A 16-day-old baby girl patient was referred to King Abdulaziz Medical City Jeddah, Saudi Arabia, due to dysmorphic features. She was a product of normal spontaneous vaginal delivery at 35 weeks of gestation with a good Appearance, Pulse, Grimace, Activity, and Respiration (APGAR) score, and a birth weight of 2.2 Kg. The antenatal history was unremarkable for the baby girl. The mother is 35 years old, and the father is 36 years old. They are second-degree relatives, and this is their first child. There is a positive family history of a child with severe short stature.

On physical examination, her length was 44 cm (below 3rd centile), her weight was 1.9 kg (below 3rd centile), and her head circumference was 29 cm (below 3rd centile).

The case vital signs were as follows: pulse, 150 beat/min; blood pressure, 97/60 mmHg; respiratory rate, 36 breath/min; and temperature, 37.2^o^C.

Physical examination revealed facial dysmorphism in form of low set ears, long philtrum, and upslanting palpebral fissures of both eyes. In addition, there was a short proximal bone in the upper limb with contracture in both elbows and talipes equinovarus. She had normal first, second heart sounds, and a pansystolic murmur. She was hypotonic; other examinations and an overall systemic review were unremarkable. Laboratory investigations for the patient are illustrated in Table 1.

**Table 1:** Laboratory investigations

Test	Value	Reference range
White blood cells	9.5×10^9^/L	( 5.0 – 19.5 ×10^9^/L)
Red blood cells	3.6 × 10^12^/L	(3.0 – 5.4 × 10^12^/L)
Hemoglobin	11.8 gm/dL	(10.1– 18 gm/dL)
Platelet	228 ×10^9^/L	(150 – 450×10^9^/L)
Random glucose	4.2 mmol/L	( 2.8 – 4.4 mmol/L)
Urea	3.9 mmol/L	( 1.2 – 6.0 mmol/L)
Potassium	6.9 mmol/L	( 3.6 – 5.8 mmol/L)
Chloride	103 mmol/L	( 101 – 111 mmol/L)
Sodium	136 mmol/L	( 135 – 144 mmol/L)

**Figure 1:**
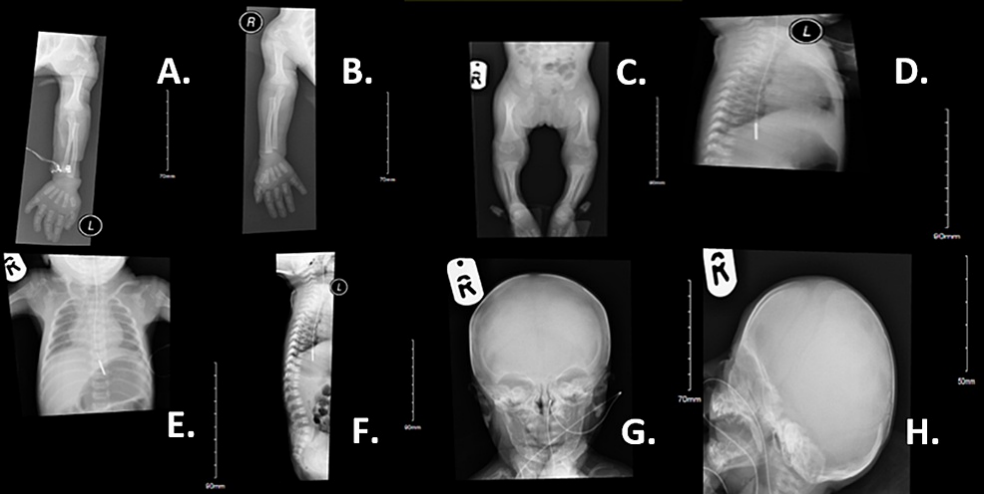
Skeletal survey The skeletal survey shows bilateral rhizomelic and shortening of the humeri and the femurs (A, B, C). Punctate ossification is identified at the proximal femur and distal femur and around the knees (C). Punctate ossification is noted at the proximal humeri bilaterally around the shoulder joint and along the upper spine (A, B, D, F). The vertebral bodies are not remarkable (F). The pelvis is within the normal limit (C). The head is within the normal limit (G, H). The nasogastric tube tip is seen just beyond the gastroesophageal junction (D, E). The stomach is distended (D, E). Normal cardio-mediastinal outline (E).

A skeletal survey showed bilateral rhizomelic and shortening of the humeri and femurs. Punctate ossification was identified at the proximal femur, distal femur, and around the knees. Moreover, punctate ossification is noted at the proximal humeri bilaterally around the shoulder joint and along the upper spine (Figure 1).

**Figure 2:**
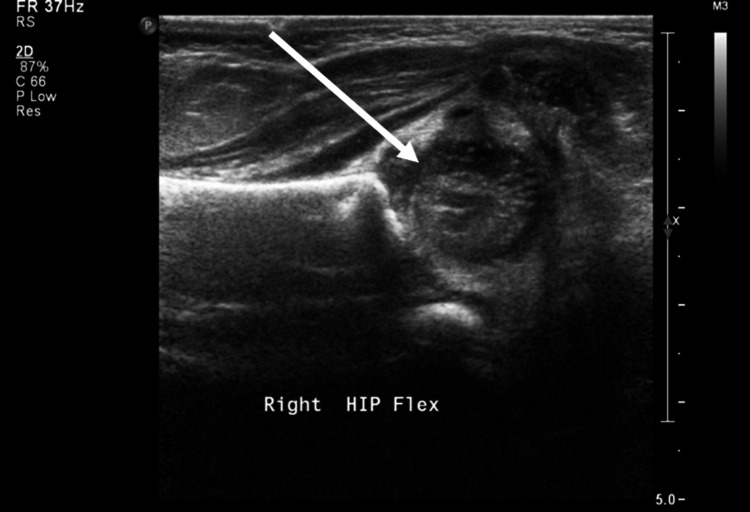
Hip ultrasound Bilateral femoral heads are dislocated (arrow).

The hip ultrasound showed femoral head dislocation on both sides (Figure 2).

**Figure 3:**
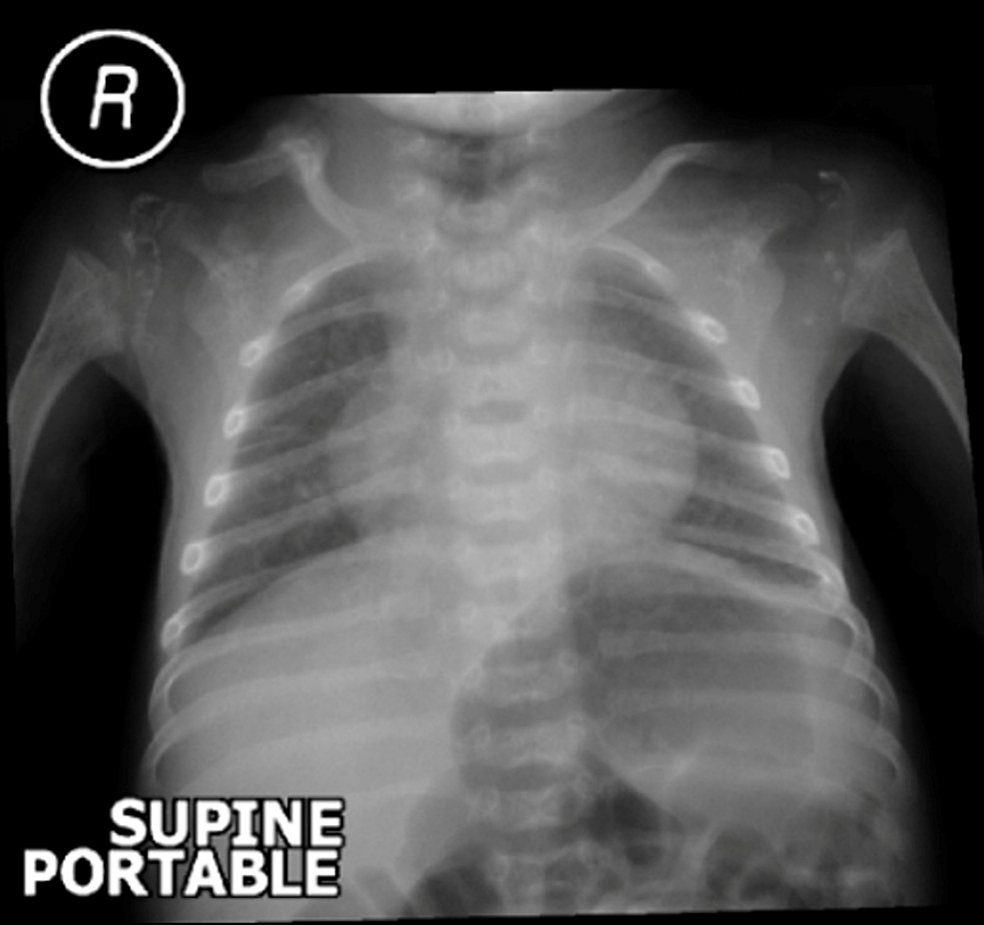
Chest radiography Chest radiography shows cardiomegaly with prominent lung markings. Airspace opacities, pleural effusion, and pneumothorax are absent.

Furthermore, a chest x-ray revealed cardiomegaly with prominent lungs markings. Radiography did not show evidence of air space opacities, pleural effusion, and pneumothorax (Figure 3).

In addition, an echocardiogram confirmed the diagnosis of tetralogy of Fallot.

Genetic testing with whole-exome sequencing (WES) revealed homozygous 4-bp deletion variant c.1639_1642del p. (Arg547*) in the *AGPS* gene, which led to a premature stop codon. This confirms the diagnosis of RCDP3. Furthermore, the genetic testing results found that both parents are heterozygous carriers for RCDP3. Therefore, upon reviewing the baby's condition and the associated medical anomalies by the multidisciplinary team, parents were counseled about the natural history of the condition and its poor prognosis.

## Discussion

Chondrodysplasia punctata (CDP) is a rare genetic disorder that includes disorders of skeletal dysplasia, which are heterogeneous in their clinical features and mode of inheritance. There are three types of CDP based on the mode of inheritance: arylsulfatase E deficiency (CDPX1), an X-linked recessive mode of inheritance; Conradi-Hünermann-Happle syndrome (CDPX2), an X-linked dominant disorder; and RCDP, which is autosomal recessively inherited [1]. Five types of RCDP are described in the literature, all of which are characterized by rhizomelia, a shortening in the proximal long bones that is symmetrical in distribution. The dysmorphic facial features may include a small nose with upturned nostrils, full cheeks, hypertelorism (widely-set eyes), and midface hypoplasia. Bilateral congenital cataracts and congenital contractures might be detected soon after birth. Later, severe growth and mental retardation prevail more [1,10,11]. Diagnosis of RCDP is made based on biochemical and/or molecular genetic testing [3]. The majority of affected individuals do not survive the first decade of life, and those who survive will live with physical disabilities and cognitive impairment [3,12].

The case reported in this article showed severe short stature. All her growth parameters were below the 3rd centile, which is consistent with one case of the same condition by Itzkovitz B et al. They reported a four-year-old girl ("patient 6" in the report) who fell below 3rd centile in all growth parameters [5]. Another seven-year-old girl ("patient 7") reported simplified ears and long philtrum [5]. This matches some of the signs observed in our case as the patient had low-set ears; however, she had a short philtrum. Limb contractures were present bilaterally in both cases. Upon skeletal survey, in the report by Itzkovitz B et al., the x-ray showed short humeri and femurs in patient 7, and rhizomelia of the upper limbs in patient 6 [5]. It is noteworthy that similar findings to both cases were observed in the present case report. Moreover, in the current case, punctata were noted at proximal humeri around the shoulders, proximal and distal femur, around the knees, and upper spine. In the article by Itzkovitz B et al., x-rays of patient 5 showed punctata at elbows, hips, knees, vertebrae, and trachea, while patient 6 showed punctata at shoulders, bilaterally [5]. Congenital cataracts were identified in all three reported cases (patients 5, 6, and 7) [5] as well as in other multiple patients in various reports [5,6,8,9]. Similarly, numerous other equal findings were reported, such as failure to thrive, mental retardation, and seizure confirmed with EEG [5,6,8,9]. Moreover, brain imaging for a few patients showed microcephaly, agenesis of the corpus callosum, and atrophy of most cerebral areas [5,6]. Interestingly, in one case reported by Noguchi M et al., the patient did not encounter visual or hearing problems [8]. Additionally, the five-year-old girl could eat and did not have difficulty swallowing; furthermore, she was able to stand with some help and could, indeed, walk independently. However, limited motion at large joints was noted [8]. The same patient presented with some characteristic features such as intrauterine growth retardation, failure to thrive, bilateral congenital cataracts, rhizomelia, joint contractures, and stippled epiphyses. Furthermore, she encountered other medical problems such as congenital diaphragmatic hernia [8]. Three patients reported by Thai TP et al. [6] suffered from hypotonia and other manifestations similar to our patient. Our patient had a bilateral femoral head dislocation, consistent with a case reported by Itzkovitz B et al. [5]. Moreover, radiographs of the case mentioned above showed kyphoscoliosis and coronal vertebral clefting [5]. It is worth noting that the 16-day-old baby girl presented to our hospital early in age. This gave us an opportunity for accurate diagnosis and subsequently a vital time to counsel the parents regarding the nature of the condition and the unfortunate prognosis associated with such a case.

## Conclusions

RCDP is a rare proximal autosomal recessive disorder. It is characterized by classical features, such as short proximal limb bones, congenital cataracts, growth and mental retardation, and dysmorphic facial features. This article reports a case of RCDP type 3, which was caused by a pathogenic frameshift variant in *AGPS*. Numerous key features of RCDP were present in this case, some of which are bilateral shortening of the humeri and femurs, and bilateral femoral head subluxation.
